# Mapping protein interactions by combining antibody affinity maturation and mass spectrometry

**DOI:** 10.1016/j.ab.2011.05.005

**Published:** 2011-10-01

**Authors:** Michael R. Dyson, Yong Zheng, Cunjie Zhang, Karen Colwill, Kritika Pershad, Brian K. Kay, Tony Pawson, John McCafferty

**Affiliations:** a Department of Biochemistry, University of Cambridge, Cambridge CB2 1QW, UK; b Samuel Lunenfeld Research Institute, Mount Sinai Hospital, Toronto, Ontario, Canada M5G 1X5; c Department of Biological Sciences, University of Illinois at Chicago, Chicago, IL 60607, USA; d Department of Molecular and Medical Genetics, University of Toronto, Toronto, Ontario, Canada M5S 1A8

**Keywords:** Antibody phage display, Affinity maturation, Immunoprecipitation, Mass spectrometry, Protein–protein interaction networks

## Abstract

Mapping protein interactions by immunoprecipitation is limited by the availability of antibodies recognizing available native epitopes within protein complexes with sufficient affinity. Here we demonstrate a scalable approach for generation of such antibodies using phage display and affinity maturation. We combined antibody variable heavy (V_H_) genes from target-specific clones (recognizing Src homology 2 (SH2) domains of LYN, VAV1, NCK1, ZAP70, PTPN11, CRK, LCK, and SHC1) with a repertoire of 10^8^ to 10^9^ new variable light (V_L_) genes. Improved binders were isolated by stringent selections from these new “chain-shuffled” libraries. We also developed a predictive 96-well immunocapture screen and found that only 12% of antibodies had sufficient affinity/epitope availability to capture endogenous target from lysates. Using antibodies of different affinities to the same epitope, we show that affinity improvement was a key determinant for success and identified a clear affinity threshold value (60 nM for SHC1) that must be breached for success in immunoprecipitation. By combining affinity capture using matured antibodies to SHC1 with mass spectrometry, we identified seven known binding partners and two known SHC1 phosphorylation sites in epidermal growth factor (EGF)-stimulated human breast cancer epithelial cells. These results demonstrate that antibodies capable of immunoprecipitation can be generated by chain shuffling, providing a scalable approach to mapping protein–protein interaction networks.

The creation of a resource of binders to all proteins in the proteome together with their splice variants and posttranslational modifications would be of great value to the research community [1,2]. Application of such antibodies to affinity capture of protein complexes (immunoprecipitation) would enable the characterization of cellular signaling networks in response to various stimuli. Current methods require the artificial “tagging” of proteins that may alter their activity and cellular location, whereas the use of binders to native proteins allows one to directly observe unmodified proteins. There are several initiatives and proposals relating to genome-wide antibody generation [1,3–5]. The ability to generate large sets of antibodies validated for use in immunoprecipitation represents an ambitious goal that would nonetheless be of enormous value to the research community in understanding protein interaction networks.

This goal can best be achieved by screening large recombinant phage [6,7], yeast [8,9], or ribosome [10] display libraries, which is a faster and more scalable approach than animal immunization. Antibody phage display, first described two decades ago [11], is a powerful method for selecting novel binders from large antibody libraries by linking the binding properties of an antibody displayed on the surface of filamentous bacteriophage to the encoding DNA within the bacteriophage. As an alternative to antibodies, other scaffolds have been used with phage display to generate binding molecules, including combinatorial peptides [12], the Z domain of protein A [13], the fibronectin type III domain [14], and designed ankyrin repeat proteins [15].

In a recent international collaboration, we and others demonstrated the feasibility of generating research reagents by phage display by targeting a group of 20 Src homology 2 (SH2)^1^ domain-containing proteins [5,16,17]. SH2 domains mediate many signal transduction processes through their ability to bind phosphorylated tyrosine (pTyr)-containing polypeptides [18,19]. In humans, there are 120 SH2 domains found within 110 distinct proteins that represent several different protein classes, including kinases, phosphatases, adapters, and transcription factors [20]. The SH2 domain consists of approximately 100 amino acids that fold into an antiparallel β-sheet flanked by two α-helices. Binding specificity of the SH2 domain is typically mediated by the 3–6 amino acids C terminal to the pTyr in the target sequence [21,22]. Given their widespread role in signal transduction and the availability of many characterized antibodies, in this study we focused on eight different SH2 domain proteins: LYN, VAV1, NCK1, ZAP70, PTPN11, CRK, LCK, and SHC1. For more detailed quantitative studies, we concentrated on the SH2 domain-containing protein SHC1. SHC1 is a prototypic scaffold protein that is essential for cancer progression [23]. It links activated receptor protein tyrosine kinases to the Ras-MAPK pathway and is a mediator of cell responsiveness to different external stimuli involving G protein-coupled receptors, immunoglobulin receptors, integrins, and non-receptor tyrosine kinases [24]. SHC1 has been shown to direct tissue morphogenesis during development [24] and encodes three splice variants of 46 kDa (p46SHC1), 52 kDa (p52SHC1), and 66 kDa (p66SHC1). Overexpression of p66SHC1 accelerates ES cell neural induction and modulates the Wnt/β-catenin pathway [25].

The availability of immunoprecipitation reagents to these and other signaling molecules would be of enormous benefit for studying signaling interactions and dynamics either individually or at a systems biology level*.* Despite the long history of the use of antibodies in immunoprecipitation, and despite the importance of the technique, there have been no studies examining the relationship between antibody affinity and performance in immunoprecipitation. Immunoprecipitation is a particularly challenging application for antibodies because it requires affinity capture and retention of native proteins and their complexes present at relatively low concentrations in cells or tissues. Given these requirements, we anticipated that high affinity would be a crucial determinant of success and sought to improve the affinity of antibodies emerging from phage display selections. Following the initial selection of antibodies recognizing SH2 domains [17], we employed “chain-shuffling” to create secondary gene-specific libraries.

In our antibody display library, antibodies are presented in the form of single chain variable fragments (scFvs), where the heavy chain variable region genes (V_H_) and light chain variable region genes (V_L_) are joined by a flexible linker peptide. Although the initial phage antibody selections [17] were performed with a very large antibody library consisting of more than 10^10^ clones [7], we reasoned that any V_H_ will have paired with a limited number of V_L_ partners (and vice versa) and that each might not have found its optimal partner from the available repertoires. Therefore, we employed chain shuffling as a simple method for creating secondary diversified libraries from individual antibodies from which we could select higher affinity variants. Because the greatest diversity resides within the V_H_ region, we retained selected V_H_ genes and shuffled these with a repertoire of V_L_ genes. Therefore, we created diversified libraries to eight targets in parallel by cloning selected mixes of V_H_ genes from primary selections [17]. These libraries were subjected to stringent selections using limiting concentrations of biotinylated antigen.

The availability of the target epitope is an important factor in determining success in immunoprecipitation. With this in mind, we developed a novel 96-well immunocapture screen to rapidly identify binders recognizing available epitopes with sufficient affinity to capture low levels of endogenous SHC1 in a breast cancer epithelial cell line. The affinities and off-rates of a panel of anti-SHC1, all sharing the same V_H_ gene, were measured, and these correlated with their ability to work in immunoprecipitation. We demonstrated the use of our anti-SHC1 antibodies in immunoprecipitation coupled to mass spectrometry (MS) to identify known binding partners of SHC1 during epidermal growth factor (EGF) signaling.

## Materials and methods

### Generation of scFv libraries by chain shuffling

The V_H_ region of selected scFvs specific to the SH2 domains (LYN, VAV1, NCK1, ZAP70, PTPN11C, CRK, LCK, and SHC1) were polymerase chain reaction (PCR) amplified from pSANG10-TEV plasmid DNA [17] with the primers pSANG10-PelB (CGCTGCCCAGCCGGCCATGG) and HLINK (ACCGCCAGAGCCACCTCCGCC). The PCR reactions consisted of primers (0.5 μM), 2× Qiagen Hot Start *Taq* PCR mix (25 μl), and plasmid DNA (100 ng) in a total volume of 50 μl. The set of amplified V_H_ genes for each target antigen were pooled and purified by Qiagen spin column, and inserts (15 μg) were digested with *Nco*I (40 U) and *Xho*I (60 U) in 200 μl of NEB buffer 2 (50 mM NaCl, 10 mM Tris–HCl [pH 7.9], 10 mM MgCl_2_, 1 mM dithiothreitol [DTT], and 0.1 mg/ml bovine serum albumin [BSA]) for 3 h at 37 °C. Digested inserts were purified by Qiagen spin column and ligated to the *Nco*I/*Xho*I cut pSANG4 vector harboring the naive V_L_ κ- and λ-chain libraries [7]. Ligation reactions contained V_H_ insert (1 μg), pSANG4 vector (2.5 μg), 50 mM Tris–HCl (pH 7.5), 10 mM MgCl_2_, 1 mM ATP, 10 mM DTT, and T4 DNA ligase (1000 U, New England Biolabs) in a total volume of 125 μl and were incubated at 16 °C for 16 h. Ligation reactions were phenol–chloroform extracted by the addition of 175 μl of 10 mM Tris–HCl (pH 8.0) to each ligation reaction, followed by extraction with 300 μl of phenol–chloroform–isoamylalcohol (25:24:1), and then vortexed and spun at 15,600*g* (2 min, 4 °C). The top aqueous layer was transferred to a fresh microfuge tube containing 280 μl of chloroform, vortexed, and spun at 15,600*g* (2 min, 4 °C), and the top aqueous layer was transferred to a fresh microfuge tube containing 1/10 volume (20 μl) of 3 M potassium acetate (pH 7.0). To this, 2.5 volumes (500 μl) of chilled 100% ethanol was added, incubated on ice for 10 min, spun at 15,600*g* (10 min, 4 °C), supernatant decanted, pellet washed with 600 μl of chilled 75% ethanol, spun at 15,600*g* (5 min, 4 °C), supernatant decanted, pellet air dried at 37 °C for 10 min, and dissolved in 4 μl of 10 mM Tris–HCl (pH 8.0). Here 2 μl of ligation reaction was used to electroporate 40 μl of electrocompetent TG1 cells in duplicate (Bio-Rad MicroPulser, EC1 channel), followed by the addition of 2 ml of SOC medium and incubation at 37 °C for 40 min. Cells were plated on 2× TY agar plates containing ampicillin (100 μg/ml) and glucose (2%). Dilutions of the transformation were also plated to determine library size, which ranged from 1.5 × 10^8^ to 2 × 10^9^ clones, with 77–93% of the transformants being positive for insertion scFv encoding insert, as determined by colony PCR.

### Phage display selections, subcloning scFv populations into an expression vector, and primary ELISA

Rescue of phage particles from the chain-shuffled libraries and two rounds of selections against the eight SH2 domains were as described previously [7] except that selections employed biotinylated antigens (0.1–100 nM) that were captured with 25 μl of streptavidin-coated Dynabeads (Invitrogen, cat. no. M-280). Selected scFv populations were subcloned en masse from the phagemid vector, pSANG4, into the expression plasmid, pSANG-TEV-3F, which expresses scFvs with a six-histidine tag, a cleavage site for tobacco etch virus (TEV) protease, and three tandem copies of the FLAG epitope at their C termini. Small-scale expression of the scFvs in *Escherichia coli* grown in 96-well plates and primary enzyme-linked immunosorbent assay (ELISA) screening has been described previously [17].

### Inhibition polyclonal phage ELISA

To compare the relative binding strengths of the scFvs, which were displayed on phage particles, an inhibition phage ELISA was performed. First, phage dilutions were incubated with antigen-coated wells to determine the optimal phage concentration to use for the inhibition assays. Black, 96-well flat-bottom, Maxisorp polystyrene plates (Nunc, cat. no. 43711) were coated with antigen (2.5 μg/ml) in phosphate-buffered saline (PBS) overnight, blocked with 2% milk powder in PBS (PBSM) for 1 h, and then incubated with serial dilutions (2×–128× in PBSM) of specific phage populations for 1 h. Plates were washed three times with PBS and 0.1% Tween 20 (PBST), washed three times with PBS, and then incubated with anti-M13 antibody (1 μg/ml in PBSM, GE Healthcare). After 1 h, plates were washed and bound phage particles were detected with a europium-labeled anti-mouse secondary antibody (Perkin–Elmer) in a DELFIA time-resolved fluorescence assay. A phage concentration, giving a signal of between 10,000 and 20,000 europium fluorescence units, was then used in the inhibition assay, where a fixed concentration of phage particles was preincubated with varying concentrations of antigen (1–1000 nM) at 4 °C in PBSM prior to incubation with antigen-coated wells for 30 min. Detection was as described above.

### scFv inhibition ELISA and IP-ELISA

scFv inhibition ELISA was used to affinity rank individual clones. scFvs were expressed in bacteria grown in 96-well plates using autoinduction media [26], and culture supernatants (diluted 2- to 60-fold in PBSM) were incubated with antigen-coated plates and detected as above to identify suitable dilutions of clone sets for inhibition assays. The scFv inhibition assay was as described above for the inhibition phage ELISA except that fixed antigen concentrations were employed (316, 100, and 31.6 nM) and detection employed a europium-labeled anti-FLAG antibody (Sigma). For immunoprecipitation ELISA (IP-ELISA), 50 μl (5 μg/ml) of anti-FLAG antibody in PBS (Sigma) was used to coat polystyrene 96-well plates overnight, followed by blocking in PBSM. After washing three times with PBST and three times with PBS, 50 μl of scFvs was added to each well (consisting of 25 μl of culture supernatant and 25 μl of 2× PBSM) and incubated for 1 h to permit capture of the scFvs via their FLAG epitope tags. Plates were washed, and then 50 μl of cell lysate (prepared as described below) was added. The plates were incubated overnight at 4 °C and washed. Bound endogenous full-length SHC1 was detected by the addition of 50 μl (0.5 μg/ml) of anti-SHC1 rabbit polyclonal antibody (BD Biosciences, cat. no. 610085) in PBSM. Subsequently, the plates were washed, followed by the addition of 50 μl (0.5 μg/ml) of europium-labeled goat anti-rabbit antibody (Perkin–Elmer).

### Expression and purification of scFvs

Single chain antibodies were initially expressed from the expression vector pSANG-TEV-3F [17] and subsequently from pSANG10-3F [27], which gives a superior expression yield. Antibodies were expressed in *E. coli* BL21(DE3) or the T-phage-resistant *E. coli* BL21(DE3) V2R pRARE2, which was kindly provided by Douglas Cossar and Peter Loppnau (Structural Genomics Consortium, Toronto, Canada). Details regarding bacterial expression of scFvs at a 500-ml scale in autoinduction media [26], periplasmic extraction, and affinity purification were as described previously [27]. Selected scFvs were expressed as Fc fusions by cloning into an Fc expression vector [28], transient transfection of HEK293E cells [29], followed by affinity purification from culture supernatants.

### BIAcore kinetic analysis

Size exclusion chromatography revealed that the purified scFvs were a mixture of monomer and dimer (data not shown). Therefore, to enable monovalent binding kinetics, scFvs were subcloned into the vector pSANG10-HC (see supplementary material), which adds a His10-CLAMP tag [30] onto the C terminus of the scFvs. Expression and purification of scFvs were as described above. The “affinity clamp” protein (ePDZ-b1) was expressed and purified as reported previously [30] and covalently attached (70 response units [RU]) to a dextran-coated CM5 sensor chip using standard EDC/NHS (*N*-ethyl-*N*′-(dimethylaminopropyl) carbodiimide/*N*-hydroxysuccinimide) chemistry [31]. The running buffer for all BIAcore experiments was filtered PBS (Dulbecco A, Oxoid, cat. no. BR0014G) supplemented with 0.05% Tween 20. Prior to each kinetic experiment, a conditioning step was performed where the regeneration buffer (10 mM glycine, pH 1.5) was injected (15 μl, 30 μl/min) over the sensor chip surface. For each kinetic cycle, scFvs (150 nM, 15 μl injection, 5 μl/min flow rate) were bound to the immobilized ePDZ-b1 via the CLAMP tag. After a 2-min stabilization period (5 μl/min flow rate), increasing concentrations of purified SHC1 (see Supplementary Fig. 1 in the supplementary material for concentration ranges) were injected (30 μl) into the BIAcore T100 at a 30-μl/min flow rate. Binding and dissociation were monitored over 60- and 120-s windows, respectively. After each cycle, 10 mM glycine (pH 1.5) was used to strip the scFvs/SHC1 off the sensor chip (15 μl, 30 μl/min). The binding profiles were evaluated using the global fitting analysis with BIAevaluation T100 software and a 1:1 interaction model.

### Immunoprecipitation of endogenous SHC1 from human breast cancer epithelial cell line MDA-MB231

Adherent MDA-MB231 cells were propagated in 145-mm tissue culture dishes in RPMI 1640 medium (PALL), which was supplemented with 5% fetal bovine serum (FBS). When cells reached 70–80% confluency, they were washed with PBS (5 ml/dish), scraped off the plate with PBS (2 ml/dish), and pelleted by centrifugation (200*g,* 4 min). Cells (∼10^7^/dish) were resuspended (2 ml/dish) in NP-40 lysis buffer (50 mM Hepes–KOH [pH 8.0], 100 mM KCl, 2 mM ethylenediaminetetraacetic acid [EDTA], 0.5% NP-40, 50 mM NaF, and 2 mM DTT), which was supplemented with protease and phosphatase inhibitors (50 mM β-glycerolphosphate, 10 μg/ml aprotinin, 10 μg/ml leupeptin, 1 mM vanadate, and 1 mM phenylmethylsulfonyl fluoride [PMSF]) and incubated on ice for 10 min. Lysates were cleared by centrifugation (25,000*g,* 20 min). FLAG-tagged anti-SHC1 scFv antibody (2 μg) was prebound to FLAG-M2 agarose (20 μl, 50% slurry, Sigma) in 400 μl of lysis buffer for 30 min. The resin was washed three times prior to the addition of 1 ml of clarified MDA-MB231 cell lysate. The cell lysate was incubated with resin for 1 h at 4 °C (by end-over-end mixing), and the resin was washed four times with 1 ml of NP-40 lysis buffer. Then 2 volumes of sodium dodecyl sulfate–polyacrylamide gel electrophoresis (SDS–PAGE) loading buffer (50 μl) was added to the washed resin. The immunoprecipitates were heated to 95 °C for 5 min and resolved by 4–20% SDS–PAGE, followed by Western blotting to polyvinylidene fluoride (PVDF) membrane. Blots were probed with rabbit polyclonal anti-SHC1 (1:2000, BD Biosciences) and goat anti-rabbit IR800 (1:15,000, LICOR). Immunoprecipitation with adherent HEK293 cell lysates was as described above except that cells were propagated in high-glucose Dulbecco’s modified Eagle’s medium (DMEM, PALL).

### Immunoprecipitation of endogenous SHC1 present in lysates of fibroblasts

Approximately 5 million Rat2 fibroblasts were stimulated with EGF (100 ng/ml) for 3 min, washed twice with cold PBS, and lysed in 1 ml of lysis buffer (50 mM Hepes [pH 8.0], 100 mM KCl, 0.5% NP-40, 10% glycerol, 0.5 mM ethyleneglycoltetraacetic acid [EGTA], 1 mM Na_3_VO_4_, 2.5 mM NaPPi, 1 mM PMSF, and protease inhibitors [Sigma]). Cell debris was removed by centrifugation (14,000*g,* 30 min). Nuclear-free lysate was subsequently incubated with rabbit polyclonal anti-SHC1 antibody (BD Biosciences) that was prebound to protein A agarose (40 μl, 10% slurry, Generon) or with FLAG-tagged anti-SHC1 scFv antibody that was prebound to FLAG-M2 agarose (50% slurry, Sigma) for 6 h. Immunoprecipitants were washed six times with lysis buffer and twice with 50 mM ammonium bicarbonate (pH 8.0), followed by on-bead tryptic digestion with 0.4 μg of trypsin (Promega) at 37 °C for 6 h. The resulting peptide mixture was transferred to a new Eppendorf tube for acidification and drying.

### MS analysis

Dried peptides were reconstituted with 5% formic acid. MS analysis was performed on a hybrid triple quadrupole/ion trap mass spectrometer (4000 Q Trap, ABI/MDS Sciex). Chromatographic separations of peptides were carried out on a nano-LC system (Eksigent) coupled to a 100-μm (i.d.) fused-silica column packed with 5 μm Zorbax C18 as the trap column and a 75-μm (i.d.) fused-silica column packed with 3.5 μm Zorbax C18 as the column. A micro-Tee and injection valves were used to connect both columns. Peptides were separated with a linear gradient from 2 to 30% acetonitrile in 90 min at a flow rate of 300 nl/min. An in-house-developed and validated multiple reaction monitoring (MRM) assay, specifically designed to target SHC1 binding partners, was used to detect SHC1 and its interacting proteins in samples. A full tandem MS (MS/MS) spectrum for each observed peptide was also acquired for sequence validation. Acquired raw files were converted to MGF format and searched against the Ensembl Rat database (release version 49) using Mascot (version 2.1). The search result in DAT format were subsequently processed with Peptide and Protein Prophet embedded in Scaffold (Proteome Software) using a protein identification threshold at more than 90% confidence.

## Results

### Creation of chain-shuffled libraries and selection

**Fig.1:**
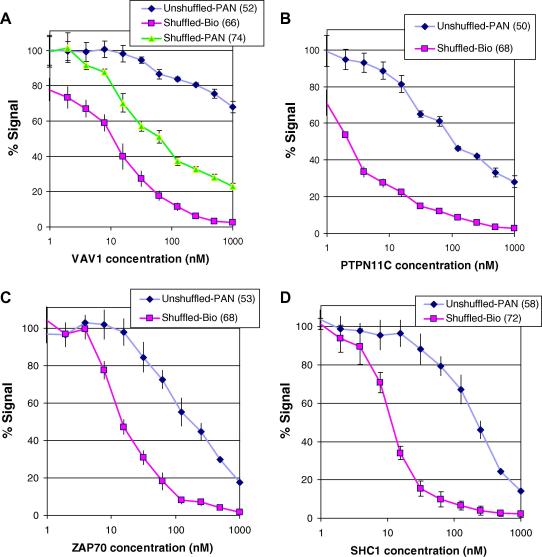
Improvement in antibody affinity following chain shuffling. Inhibition ELISAs were conducted by preincubating polyclonal phage derived from two rounds of selection with a range of antigen concentrations prior to incubation on antigen-coated wells. Bound phage populations were detected with anti-M13 antibody. Signal in ELISA from these samples is expressed relative to the uninhibited control. Phage particles were derived from panning using either the original library (“Unshuffled-PAN”) or the shuffled library (“Shuffled-PAN”) or by stringent selection on biotinylated antigen (“Shuffled-Bio”). Examples are shown for the SH2 domain target VAV1 (A), the C-terminal domain of PTPN11C (B), and the ZAP70 tandem (C) and SHC1 (D) SH2 domains. Selection numbers, which are also used in clone names, are in parentheses.

**Fig.2:**
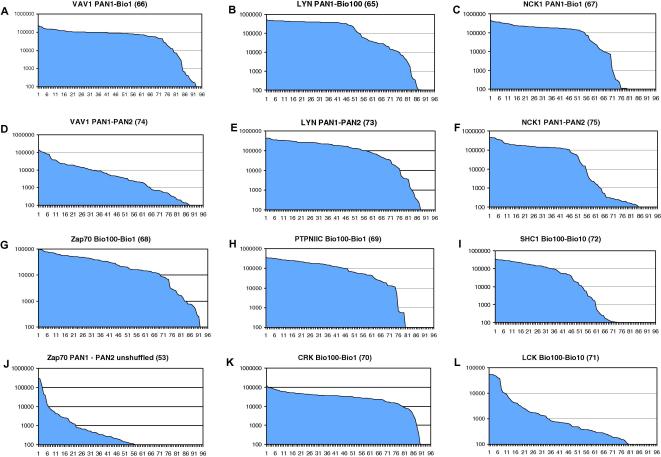
Primary ELISA screen of chain-shuffled antibodies selected using each of the eight different SH2 domains. Affinity selections against the different SH2 domains with the eight chain-shuffled libraries took place under different conditions, and the optimal selections were determined based on polyclonal ELISA results (data not shown). Populations were subcloned into an optimized expression vector, and 96 clones were picked and screened by ELISA. Binding of the scFvs to the immobilized SH2 domain was quantified using europium-labeled anti-FLAG secondary antibody. A graph is shown for each of the 12 selections (A–L), plotting the time-resolved fluorescence signal in intensity units (*y* axis) for each scFv (*x* axis). The graph titles indicate the selection conditions employed (e.g., PAN1–PAN2 indicates two rounds of selections were performed with antigen immobilized on polystyrene wells, Bio100–Bio10 indicates that the first selection round was performed with 100 nM biotinylated antigen and the second round was performed with 10 nM biotinylated antigen). The selection numbers, used to name the antibodies, are shown in parentheses.

Previous selections on the SH2 domains of LYN, VAV1, NCK1, ZAP70 (tandem domains), PTPN11C (the C-terminal domain of PTPN11), CRK, LCK, and SHC1 [17] identified between 15 and 43 clones per antigen that bound only to their appropriate antigen among a set of 20 SH2 domains. V_H_ genes were PCR amplified from these and combined in antigen-specific oligoclonal mixes, and shuffled libraries were prepared by cloning the products into a phage display vector harboring the V_L_ population used in the construction of the original phage display library [7]. Chain-shuffled libraries, typically containing more than 1 × 10^9^ clones, were affinity selected under increasing stringency using limiting amounts of biotinylated antigens in solution [7]. The affinities of the selected populations were approximated using polyclonal phage in inhibition ELISA experiments (Fig. 1). A significant improvement in average affinity of the antibody populations was achieved from stringent selection of the chain-shuffled library using soluble biotinylated antigen compared with selection on the original naive phage display library (unshuffled library) carried out by “panning” on immobilized antigen [17]. For comparison, selection of the shuffled library was also carried out by panning on directly immobilized antigen in some cases. Selection under these lower stringency conditions also led to an improvement in average affinity compared with the original unshuffled selections. The best results, however, were obtained from the combination of the shuffled library and stringent selection conditions with soluble antigen, as illustrated for VAV1 (Fig. 1A). This is also reflected in the monoclonal ELISA results for VAV1 represented in Fig. 2A and D.

### Screening for high-affinity anti-SH2 scFvs

For monoclonal screening, the inserts from the selected population of phage particles were subcloned into a plasmid vector optimized for soluble antibody expression [27] and transformed into *E. coli* BL21(DE3), and 96 clones were picked for evaluation by ELISA (Fig. 2). The primary ELISA screens for all eight SH2 domains demonstrated improved results using the chain-shuffled libraries (Fig. 2) compared with the original selections using the unshuffled library [17]. For example, the proportion of clones exceeding an ELISA signal of 10,000 U increased from 6 to 69 for the ZAP70 selection using the shuffled library (Fig. 2G and J). Because the signal levels in ELISA are a function of both the expression level of the antibody and its binding strength for the target antigen, inhibition ELISAs [32] were performed with purified scFvs to help identify the highest affinity clones for each of the eight SH2 domains (data not shown). Selected scFvs were then expressed in *E. coli,* and periplasmic extracts were prepared and affinity purified. Surface plasmon resononance (SPR) measurements indicated that affinities in the low-nanomolar (nM) range were achieved [1].

Sequence analysis revealed that the chain-shuffling process had successfully generated new clones by partnering V_H_ chains with new V_L_ chains, as summarized in Table 1. (Each selection is given a number, and clones derived from that selection inherit the selection number as the first part of their name; e.g., clone 68_C01 is derived from selection 68 on ZAP70.) The greatest diversity occurs within the CDR3 sequences of heavy and light chains and for simplicity clones are grouped according to these sequences. For example, the ZAP70 selection (selection 68) resulted in 11 original V_H_ chains now partnered with new V_L_ chains to give a total of 40 unique scFv sequences (from 48 sequenced). Similarly, affinity maturation of PTPN11C binders (selection 69) resulted in a total of 6 V_H_ chains now partnered with 27 different V_L_ chains. Selection on LYN (selection 65) resulted in a total of 4 V_H_ chains now partnered with 10 different V_L_ chains. The selections for VAV (selection 66), NCK1 (selection 67), CRK (selection 70), and LCK (selection 71) resulted in only 1 or 2 different V_H_ chains partnered with new V_L_ chains.

To generate a large number of clones to analyze the relationship between immunoprecipitation success and affinity, we focussed on SHC1. ELISAs were performed on 960 clones derived from the chain-shuffled SHC library, and 607 were found to be positive in ELISA. Of these, 155 clones with the strongest ELISA signals were sequenced. Although 15 different V_H_ genes were used in the shuffled library construction, sequencing revealed that only 5 of these were represented in the top 155 clones (see Supplementary Table 1 in supplementary material). Diversity had been introduced by partnering these 5 V_H_ genes with 59 different V_L_ chains based on V_L_ CDR3 sequence. The anti-SHC1 scFvs were subdivided into “clans” sharing the same V_H_ gene. Clans 1–5 contained 1, 2, 9, 19, and 28 different V_L_ chains, respectively.

### Identification of anti-SHC1 antibodies capable of affinity capture

**Fig.3:**
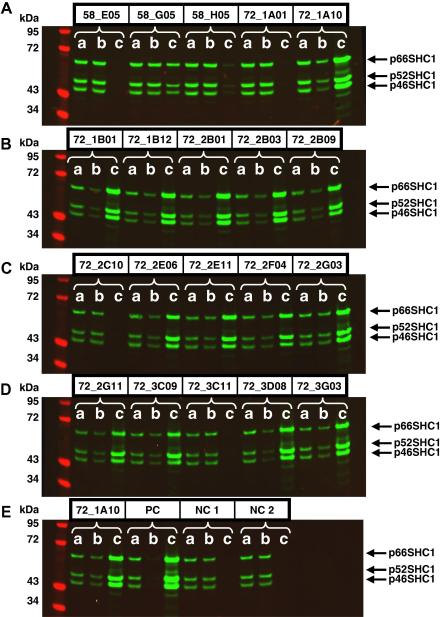
(A–E) Immunoprecipitation of SHC1 using primary and chain-shuffled antibodies. Immunoprecipitation of the three isoforms of SHC1 (66, 52, and 46 kDa) from the human breast cancer cell MDA-MB231 [33] was demonstrated using a panel of 20 unique scFvs. Immunoprecipitation was performed using anti-FLAG agarose beads (see Materials and methods), and samples were separated by SDS–PAGE, Western blotted, and detected by polyclonal anti-SHC1 antibody. Lanes a: preimmunoprecipitation lysate; lanes b: postimmunoprecipitation lysate; lanes c: immunoprecipitated sample. Individual scFvs are named according to their selection numbers and 96-well plate positions (e.g., 72_1A10 corresponds to selection number 72, plate 1, well A10). All parental scFvs have the prefix 58, and the affinity matured scFvs have the prefix 72. PC, immunoprecipitation using polyclonal rabbit anti-SHC1 antibody and protein G Sepharose; NC 1, no-antibody negative control employing anti-FLAG agarose; NC 2, no-antibody negative control employing protein G Sepharose.

Success in immunoprecipitation is likely to be determined both by antibody affinity and the availability of the recognized epitope in complexes of native protein. To examine the relationship between affinity and the potential for immunoprecipitation, a simple 96-well immunocapture assay was designed to identify antibodies capable of capturing SHC1 from lysates of the breast cancer epithelial cell line MDA-MB231 [33]. The assay involved capture of the scFvs via an immobilized anti-FLAG antibody, followed by capture of native SHC1 from MDA-MB231 lysates detected using a polyclonal antibody. Using this assay format, the top 155 binders raised against SHC1 were screened, and 19 (12%) were found to capture endogenous SHC1 from cell lysates (see Table 2, last column, for example signal intensities). A representative set of 20 positive and negative antibodies was then purified by immobilized metal affinity chromatography and used for immunoprecipitation, followed by quantitative Western blotting (see Fig. 3). There was an excellent correlation between the results of the immunocapture assay and immunoprecipitation/Western blotting assays; all positive scFv clones from the former were also strongly positive in the latter, demonstrating the value of the plate-based assay for assessing large numbers of clones.

### Efficiency of dimeric scFv–Fc fusions on immunoprecipitation efficiency

**Fig.4:**
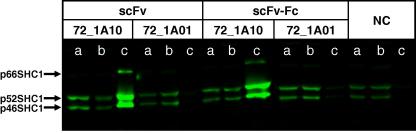
Immunoprecipitation of SHC1 with scFv or scFv–Fc fusions. Immunoprecipitation of SHC1 from HEK293 cell lysate was as described in Fig. 3 except that scFv or dimeric scFv–Fc fusions were employed. Both formats contained a C-terminal FLAG tag, enabling pull-down with anti-FLAG agarose. NC, no-antibody negative control.

To study whether dimerizing the scFvs would result in increased efficiency in pulling down endogenous SHC1 from a cell lysate, anti-SHC1 scFvs 72_1A01 and 72_1A10 were cloned into an Fc expression plasmid and, expressed in HEK293E suspension cells [28], and affinity purified. Similar results in immunoprecipitation efficiency were observed for both the mainly monomeric scFvs and dimeric scFv–Fc fusions; clone 72_1A10 efficiently pulled down endogenous SHC1, whereas clone 72_1A01 did not (Fig. 4). If the target antigen is monomeric and in solution, increasing the valency of the scFvs would not be expected to give an advantage and efficiency of capture would be based on the monovalent binding affinity. Increasing valency could be advantageous, however, if the target to be pulled down naturally existed as a multimeric complex. All pull-down experiments described here employed antibody preconjugated to resin, potentially creating a multivalent surface and an avidity effect even when the original antibody is monovalent. Although evidence is lacking for the existence of SHC1 dimers or multimers, it is known that EGF stimulation leads to a microclustering of EGF receptor (EGFR) and, by implication, its signaling complex [34]. Therefore, multivalent binding reagents may be expected to be more efficient than monovalent reagents for immunoprecipitation of the SHC1 signaling complex.

### Relationship among affinity, off-rate, and utility in immunoprecipitation

**Fig.5:**
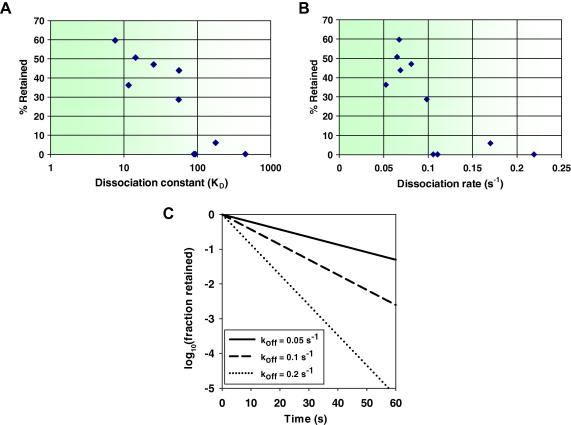
Relationship between affinity or off-rate and immunoprecipitation efficiency. Percentage recovery of target SHC1 from lysates was calculated using fluorescent Western blots. (A and B) Plots are shown of percentage SHC1 recovered against scFv dissociation constant (A) and off-rate (B). (C) Simulation plot of log fraction bound against time for different off-rates, assuming monovalent dissociation and no rebinding (http://www.graphpad.com/curvefit/dissociation_htm). Curves were fitted to the equation *y* = *e*^−^*^kt^*, where *y* is fraction bound, *k* is dissociation rate (s^−1^), *e* = 2.718, and *t* is time (s) [35]. The mathematical constant *e* is defined as the value of the derivative (slope of the tangent line) of the exponential function *f*(*x*) = *e^x^* at the point *x* = 0 and *f*(*x*) = 1.

The availability of related antibodies within a clan that share the same heavy chain variable domain provides an opportunity to examine in isolation the effect of affinity on performance within a single epitope group. Affinities were determined for 10 anti-SHC1 scFvs by SPR, including seven antibodies sharing a single V_H_ gene (see Supplementary Table 2 for full sequence alignment). A clear correlation was observed between the affinity/dissociation rate and efficiency in capturing a target from a cell lysate by immunoprecipitation. For example, the parental anti-SHC1 scFv clone 58_E05 failed in immunoprecipitation (Fig. 3A) but was surpassed by a chain-shuffled derivative 72_2B01 (Fig. 3B) with a 59-fold improvement in affinity and a 3.2-fold reduction in off-rate (Table 2). Of the eight chain-shuffled derivatives, six were positive in immunoprecipitation experiments and these had *K*_D_ values below 60 nM and off-rates of less than 0.1 s^−1^. There appears to be a relatively steep cutoff for affinity/off-rate in determining success and failure in immunoprecipitation experiments (Fig. 5A and B). One explanation is that extensive wash steps are performed to remove nonspecific binders, prior to analysis, during a typical immunoprecipitation experiment. The proportion of complex remaining over time was modeled using three different off-rates and assuming a simple exponential decay curve [35]. Exponential equations are commonly used to model the processes of radioactive isotope decay or of a ligand dissociating from its receptor. In the latter case, the rate of dissociation is proportional to the initial concentration. We adopted this exponential model to simulate the dissociation of an antibody from its target antigen. Fig. 5C shows on a logarithmic scale that a relatively modest change in off-rate can have a dramatic effect, ranging over many orders of magnitude, on the proportion of target that remains associated with the capture antibody at different time points, providing an explanation for the sharp cutoff in the relationship between affinity and immunoprecipitation success.

### Use of affinity matured anti-SHC1 antibodies to map the EGFR/SHC1 signaling complex

The utility of antibodies in capturing endogenous proteins and identifying associated binding partners was evaluated by immunoprecipitation, followed by liquid chromatograph–mass spectrometry (IP/LC–MS) analysis. Multiprotein complexes mediated by SHC1 form an essential signaling network module downstream of the activated EGFR tyrosine kinase. To identify SHC1-interacting partners, EGF-stimulated Rat2 fibroblasts were lysed and endogenous SHC1 was affinity captured with antibody 072_1A10. The recovery of SHC1 and its binding partners was then investigated using an MRM-based MS method employing procedures that we recently developed to analyze such SHC1 complexes unpublished data: Y.Z, C.Z, K.C, and T.P. This specific method allows MRM-initiated detection and sequencing (MIDAS) [36] for sequence validation, providing high confidence detection of specified interactors. In this case, a subset of seven SHC1-interacting partners was identified, comprising a core multiprotein signaling network nucleated around SHC1 during EGF signaling. We observed all seven interacting partners (EGFR, GRB2, SOS1, GAB1, PTPN11, PTPN12, and AP2) as well as two known SHC1 phosphorylation sites (pSer29 and pTyr313) (Supplementary Fig. 3 and Supplementary Table 3). A total of 34 binary interactions for human SHC1, identified in immunoprecipitation experiments, are listed in the IntAct database [37], including those listed above. In this study, we may have identified a subset of SHC1 interactions that occur relatively early following activation of the EGFR signaling pathway. Based on our quantitative analysis of the Shc1 signaling network unpublished data: Y.Z, C.Z, K.C, and T.P. the proteins identified in this article represent the most abundant and stable interactors of SHC1 downstream of EGFR signaling. In addition, the anti-SHC1 scFvs are generated against the SH2 domain of SHC1 and, thus, may interfere with some protein–protein interactions that require the SHC1 SH2 domain. Finally, the known protein–protein interactions mediated by SHC1 are pathway and cell type specific. For example, nPKCδ was identified as an SHC1 interactor in the IntAct database, but only in the context of Bcr-Abl signaling in CML K562 cells [38].

## Discussion

The combination of antibody affinity capture coupled with MS provides a powerful platform for mapping protein–protein interactions. The widespread use of this technique, however, is limited by the availability of suitable antibodies. Understanding the requirements for immunoprecipitation and increasing the availability of such reagents would be of huge advantage in mapping protein interaction networks, particularly if this could be applied in a high-throughput manner. Several factors, including the properties of the targeted epitope, determine the utility of individual antibodies for immunoprecipitation. In techniques such as Western blotting and immunohistochemistry, the antibody must recognize an epitope, typically linear, that survives the denaturation conditions used. In contrast, immunoprecipitation requires antibodies that recognize the native proteins. Depending on the supramolecular architecture of the protein complex, some epitopes will not be accessible for antibody capture because they will be occluded by other interacting proteins within the complex. Finally, affinity is also important because the method requires capture of endogenous protein present at low levels in cell lysates and also retention during the extensive wash steps employed after the complex has been captured.

Ideally, the properties of high affinity and recognition of native complexed antigen in lysates should be selected during antibody generation. Phage display provides a simple means of generating antibodies through panning of large antibody libraries on native immobilized antigen. In a recent study, we demonstrated that we could generate specific antibodies to 20 SH2 domains. In addition to demonstrating the potential for high-throughput selection, antibodies generated in this earlier work [17] provided the basis for the affinity maturation work described here.

We reasoned that the affinity of the antibody for its target would be a key parameter for success. To improve the affinity of clones emerging from phage display, we sought to use a scalable method of affinity maturation capable of dealing with multiple targets and multiple clones within a group of antibodies. In general, affinity maturation involves diversification of a lead clone followed by stringent selection from the resultant library of related binders. For diversification, the method of chain shuffling is ideally suited not just for single clones but also for oligoclonal mixes as well as whole populations. This approach was first described for affinity maturing antibodies to haptens [39–41]. Here we demonstrate that chain shuffling is a very effective way to affinity mature several antibody populations simultaneously to multiple protein targets.

Selected V_H_ genes that bound only their target antigen from a set of 20 SH2 domains were recombined with the population of V_L_ genes used in the initial construction of the library. In particular, V_H_ fragments specific to each of the eight SH2 domains were combined into oligoclonal mixes for each specific antigen and crossed with the library of V_L_ genes used in the construction of the original naive library [7], creating eight gene-specific libraries of 10^8^–10^9^ clones. After two rounds of selection with chain-shuffled sublibraries, phage inhibition ELISA showed that the average affinity of the population had improved by 1–2 orders of magnitude to the target antigen. The degree of affinity enrichment was dependent on the conditions used for the panning. In general, a first-round low-stringency panning (either antigen coated directly on a polystyrene surface or 100 nM biotinylated antigen captured on streptavidin beads) was performed first to ensure capture and amplification of the majority of binders. This was followed by a second-round selection using lower concentrations of biotinylated antigen to increase stringency. As an alternative, a strategy of “off-rate selection” could be employed [42] to select clones with a slower off-rate. The process of diversification and stringent selection led to an improvement in average affinity as measured by polyclonal ELISA using selected phage populations. After expression and purification, many individual scFvs displayed dissociation constants (*K*_D_ values) in the low nanomolar range [1].

Sequence analysis of the chain-shuffled and reselected scFvs showed that new light chain partners had been identified following stringent selection. In some cases, the representation of input V_H_ genes was reduced compared with the input library. For example, for the SHC1 selection, only 5 of the 15 different V_H_ genes used in the library appeared among 155 of the top-ranked clones. Presumably, derivatives from the other 10 V_H_ genes used in the shuffled library construction could not compete with the progeny from the top 5 V_H_ genes. A large number of novel partners were found to complement these 5 V_H_ genes. Among the 155 clones examined, the 5 V_H_ genes were found partnered with 59 different V_L_ genes (using analysis of CDR3 sequence only).

Success in immunoprecipitation requires antibodies that target epitopes that are available in native protein complexes. To focus on antibodies with this property, we developed a 96-well plate-based affinity capture screen that enabled the screening of hundreds of clones simultaneously for their ability to bind endogenous full-length SHC1 from a breast cancer epithelial cell lysate. This involved the directional immobilization of scFvs from crude culture supernatants onto 96-well plates followed by incubation with cell lysate. Captured SHC1 was detected with a rabbit anti-SHC1 polyclonal antibody. There was a strong correlation between success in the high-throughput affinity capture assay and the ability of an antibody to perform in immunoprecipitation. The assay could be used for other targets provided that a polyclonal detecting antibody was available. Potentially, the selected polyclonal phage population from which the test antibody arose could itself suffice for detection of captured target. It is interesting to note that only a minority (12%) of affinity matured scFvs were successful in the affinity capture assay, and this is likely due to a combined requirement for affinity and the need to recognize available epitopes on native protein.

Each group of antibodies sharing a V_H_ gene partnered with different V_L_ genes can be regarded as a clan that is likely to share a common epitope (especially given that the target antigen is only 110 amino acids on average), giving the opportunity to examine the effect of affinity on immunoprecipitation independent of epitope. A relationship was observed between immunoprecipitation success and affinity, as shown by selecting 10 anti-SHC1 scFvs and measuring their on- and off-rates by SPR. For both clan 3 and clan 5 (see Supplementary Table 1), there are examples where the original unshuffled clones failed or were inefficient in immunoprecipitation, but affinity maturation by chain shuffling produced higher affinity clones capable of affinity capture of SHC1 from lysates. Plotting the proportion of SHC1 captured in immunoprecipitation, quantitated from fluorescent Western blot against either equilibrium dissociation constant (*K*_D_) or dissociation rate (*k*_off_)**,** revealed a direct relationship with a sharp cutoff between success and failure in immunoprecipitation. For clan 5, the cutoff was a *K*_D_ of less than 60 nM and a *k*_off_ of less than 0.1 s^−1^. This can partly be explained by the long wash steps employed during an immunoprecipitation experiment, and as shown in Fig. 5C (modeled assuming monovalent binding and a simple exponential decay curve), small changes in the off-rate can have large effects on the proportion of target that remains associated with the capture antibody. The situation may be more complex where proteins are part of a multisubunit signaling complex, avidity effects lengthen the antibody/antigen half-life, and rebinding occurs [42], but the beneficial effect of increased off-rate is nonetheless clear.

For clans 1, 2, 3, and 5, none of the parental clones was reselected following shuffling. However, parental clones did appear in selection 72 for clan 4. Here 14 of 41 clones were parental, as judged by having an identical V_L_ CDR3 sequence. This included clone 72_1A10, which displayed good affinity for SHC1 (*K*_D_ = 25 nM) and was used successfully in immunoprecipitation. For this clan, it is clear that good antibodies were already identified from the primary selection. However, for clans 3 and 5, it is also clear that affinity maturation by chain shuffling was an absolute requirement to transform an antibody with low affinity and a failure (or poor performer) as an immunoprecipitation reagent into a higher affinity antibody that was competent to pull down endogenous levels of a target protein. Clans 1 and 2 are examples where chain shuffling and reselection failed to result in immunoprecipitation competent antibodies. Depending on the diversity of the initial phage antibody library, and on the presence of antibodies in that library that are able to bind a given epitope with sufficient affinity for endogenous immunoprecipitation, there is a strong argument to perform the population-based affinity maturation by chain shuffling described in this article both to increase the probability of identifying useful antibodies and to increase the number of immunoprecipitation competent antibodies able to bind to different epitopes.

## Conclusion

We have presented a scalable method for antibody affinity maturation, based on chain shuffling, to generate improved phage antibodies to a set of eight SH2 domain proteins. For each antigen, we matured oligoclonal mixes of clones that previously had been shown to have exquisite specificity for the target antigen, and we demonstrated that it is possible to rapidly and simultaneously affinity mature multiple specific antibodies to multiple different antigens. Using an affinity capture screen, we found that only a minority of affinity matured scFvs were successful in the capture of target antigens from lysates, and this is likely due to a combined requirement for affinity and the need to recognize available epitopes on native protein. Affinity improvements were a key determinant in the success of immunoprecipitation demonstrated using panels of antibodies with differing affinities to the same epitope. This systematic approach not only illustrated the importance of affinity but also identified a relatively sharp cutoff distinguishing between success and failure. Finally, we validated this approach by combining affinity capture using matured antibodies with MS to identify seven binding partners of SHC1 in EGF-stimulated cells. This approach, combined with MS, will help to advance our knowledge and understanding of protein interactions occurring during cell signaling.

## Figures and Tables

**Table 1:** Selection summary.

Selection number	Target	Number of input V_H_ genes	Number of V_H_ genes used	Number of unique V_L_ genes and number sequenced	Number of output V_L_ genes (based on unique V_L_ CDR3)
65	LYN	7	4	28 (29)	10
66	VAV1	6	1	30 (48)	9
67	NCK1	6	2	26 (44)	4
68	ZAP70	20	11	40 (48)	26
69	PTPN11C	12	6	42 (47)	27
70	CRK	1	1	33 (45)	7
71	LCK	12	2	22 (24)	18
72	SHC1	12	5	112 (155)	59

*Note:* Numbers sequenced are in parentheses.

**Table 2:** BIAcore kinetic data and quantitative data from affinity capture assay and quantitative Western blots showing percentage recovery in immunoprecipitation.

*Note:* Antibody names highlighted in green or red passed or failed immunoprecipitation, respectively. Parental scFvs have the prefix 58, and affinity matured scFvs have the prefix 72. Global fits were performed on the full 60 s of the association phase and the initial 20 s of the dissociation phase (see Supplementary Fig. 1). The last column (IP-ELISA) shows the signals obtained in the immunoprecipitation ELISA (see Materials and methods), which measures the ability of immobilized scFvs to bind endogenous SHC1 from a breast cancer epithelial cell lysate. (For interpretation of the references to color in this table note, the reader is referred to the Web version of this article.)

## Appendix A Supplementary data

Supplementary data 1 Surface Plasmon resonance (SPR) trace binding curves for SHC1 binding to immobilised scFv (see Experimental Procedures). Experimental data is shown as coloured lines representing different injected concentrations of SHC1. The results of a global fitting analysis with BIAevaluation T100 software and a 1:1 interaction model are shown by the black lines. Each scFv employed different sets of SHC1 injection concentrations. For 58-E05, 72-1A01, 72-2C10, 72-2B09 and 58-G05 the SHC1 concentration range was 1000, 333, 111, 37∗, 12.3, 4.1 and 1.37 nM. For 72-G03 and 72-1A10 the concentration range was 200, 66.6, 22.2, 7.4∗, 2.46, 0.82 and 0.274 nM. For 72-2B01 the concentration range was 125, 41.6, 13.9, 4.63∗, 1.54, 0.513 and 0.171 nM. For 72-2E06 the concentration range was 500, 166.5, 55.5, 18.5∗, 6.15, 2.05 and 0.685 nM. For 72-2F04 the concentration range was 166.7, 55.5, 18.5, 6.17∗, 2.05, 0.68 and 0.23 nM. Concentrations marked with an asterix were performed in duplicate. Concentration ranges were chosen to ideally be 10-fold above and below an estimated KD approximated after a single 100 nM injection of SHC1.

Supplementary data 2 Representative MS / MS spectra from selected peptides of SHC1 and its binding partners are shown. Corresponding peptide sequences, m/z values, and charge states are listed for each spectrum.

Supplementary data 3 Affinity maturation improves affinity capture, allowing detection of a core SHC1 signalling network. On-bead tryptic digestion and mass spectrometry analysis was carried out following immunoprecipitation from EGF-stimulated Rat2 fibroblasts, with the anti-SHC1 scFv 72_1A10. The identified members of the SHC1-mediated signalling complex are shown. See **Supplementary Figure 2** for representative MS/MS spectra. Phosphorylated amino acids at positions 29 (S29) and 313 (Y313) of SHC1 were also detected and shown as red dots.

Supplementary data 4 A. SHC1 – Sequence analysis of primary clones (Selection 58)

Supplementary data 5 Sequences of selected anti-SHC1 scFv

Supplementary data 6 List of identified peptides for SHC1 and its binding proteins in anti-SHC1 scFv immunoprecipitation experiments

Supplementary data 7 A minimum information about a protein affinity reagent (MIAPAR)[1] compliant document for anti-SHC1 single chain antibody scFv 72_1A10

## Footnotes

1 *Abbreviations used:* SH2, Src homology 2; pTyr, phosphorylated tyrosine; scFv, single chain variable fragment; V_H_, variable heavy; V_L_, variable light; MS, mass spectrometry; EGF, epidermal growth factor; PCR, polymerase chain reaction; DTT, dithiothreitol; BSA, bovine serum albumin; ELISA, enzyme-linked immunosorbent assay; PBS, phosphate-buffered saline; PBSM, 2% milk powder in PBS; PBST, PBS and 0.1% Tween 20; IP-ELISA, immunoprecipitation ELISA; RU, response units; PMSF, phenylmethylsulfonyl fluoride; SDS–PAGE, sodium dodecyl sulfate–polyacrylamide gel electrophoresis; MRM, multiple reaction monitoring; SPR, surface plasmon resonance; EGFR, EGF receptor.
