# Supplementary figures and images for: Mapping protein interactions by combining antibody affinity maturation and mass spectrometry
Source: Anal Biochem. 2011 Oct 1;417(1):25–35. doi: 10.1016/j.ab.2011.05.005 (PMC3171153; doi:10.1016/j.ab.2011.05.005)

## Slide 1
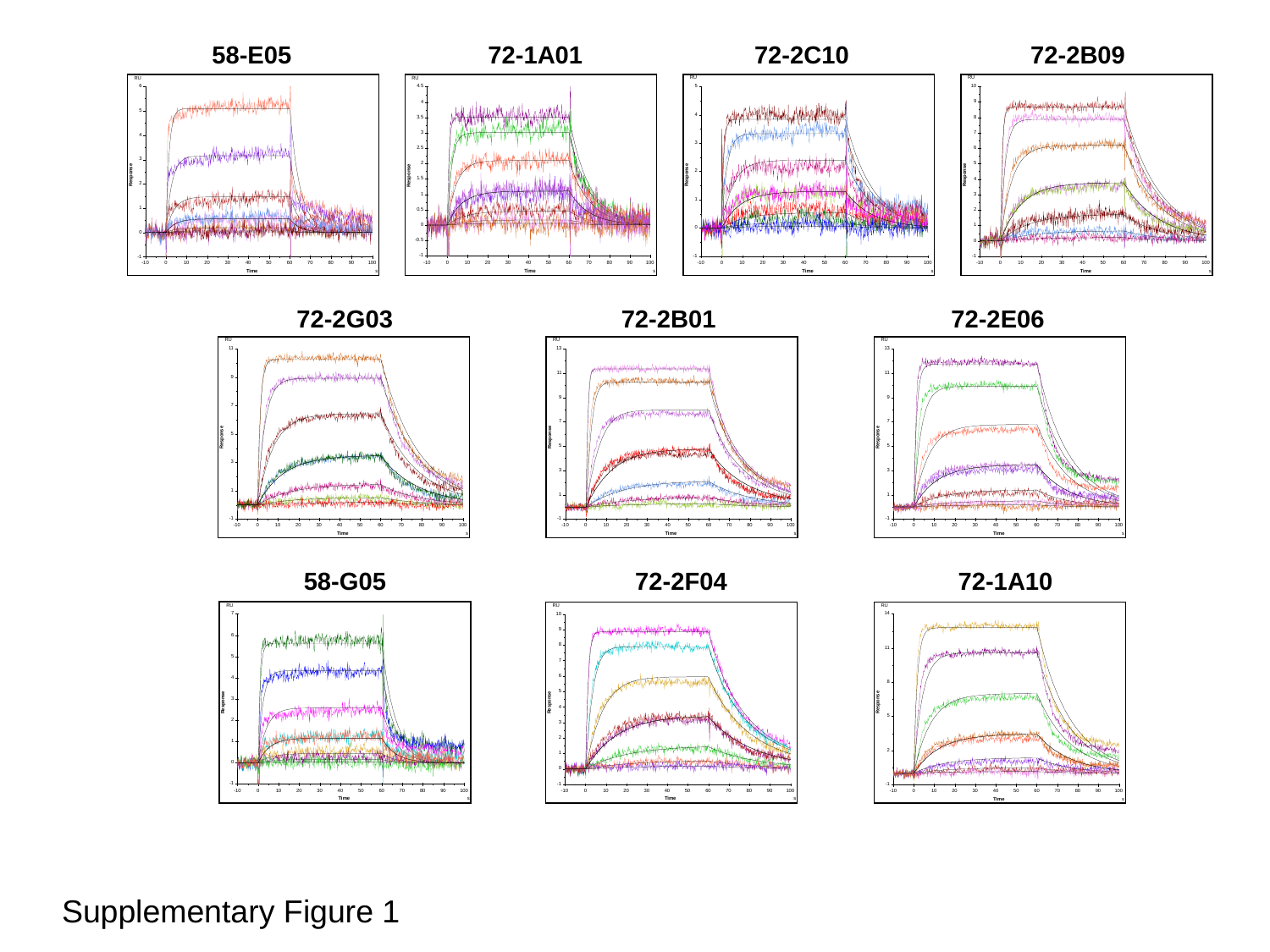

58-E05
72-1A01
72-2C10
72-2B09
72-2G03
72-2B01
72-2E06
58-G05
72-2F04
72-1A10
Supplementary Figure 1

Supplement: Supplementary data 1 — Surface Plasmon resonance (SPR) trace binding curves for SHC1 binding to immobilised scFv (see Experimental Procedures). Experimental data is shown as coloured lines representing different injected concentrations of SHC1. The results of a global fitting analysis with BIAevaluation T100 software and a 1:1 interaction model are shown by the black lines. Each scFv employed different sets of SHC1 injection concentrations. For 58-E05, 72-1A01, 72-2C10, 72-2B09 and 58-G05 the SHC1 concentration range was 1000, 333, 111, 37∗, 12.3, 4.1 and 1.37 nM. For 72-G03 and 72-1A10 the concentration range was 200, 66.6, 22.2, 7.4∗, 2.46, 0.82 and 0.274 nM. For 72-2B01 the concentration range was 125, 41.6, 13.9, 4.63∗, 1.54, 0.513 and 0.171 nM. For 72-2E06 the concentration range was 500, 166.5, 55.5, 18.5∗, 6.15, 2.05 and 0.685 nM. For 72-2F04 the concentration range was 166.7, 55.5, 18.5, 6.17∗, 2.05, 0.68 and 0.23 nM. Concentrations marked with an asterix were performed in duplicate. Concentration ranges were chosen to ideally be 10-fold above and below an estimated KD approximated after a single 100 nM injection of SHC1. [file mmc1.ppt]

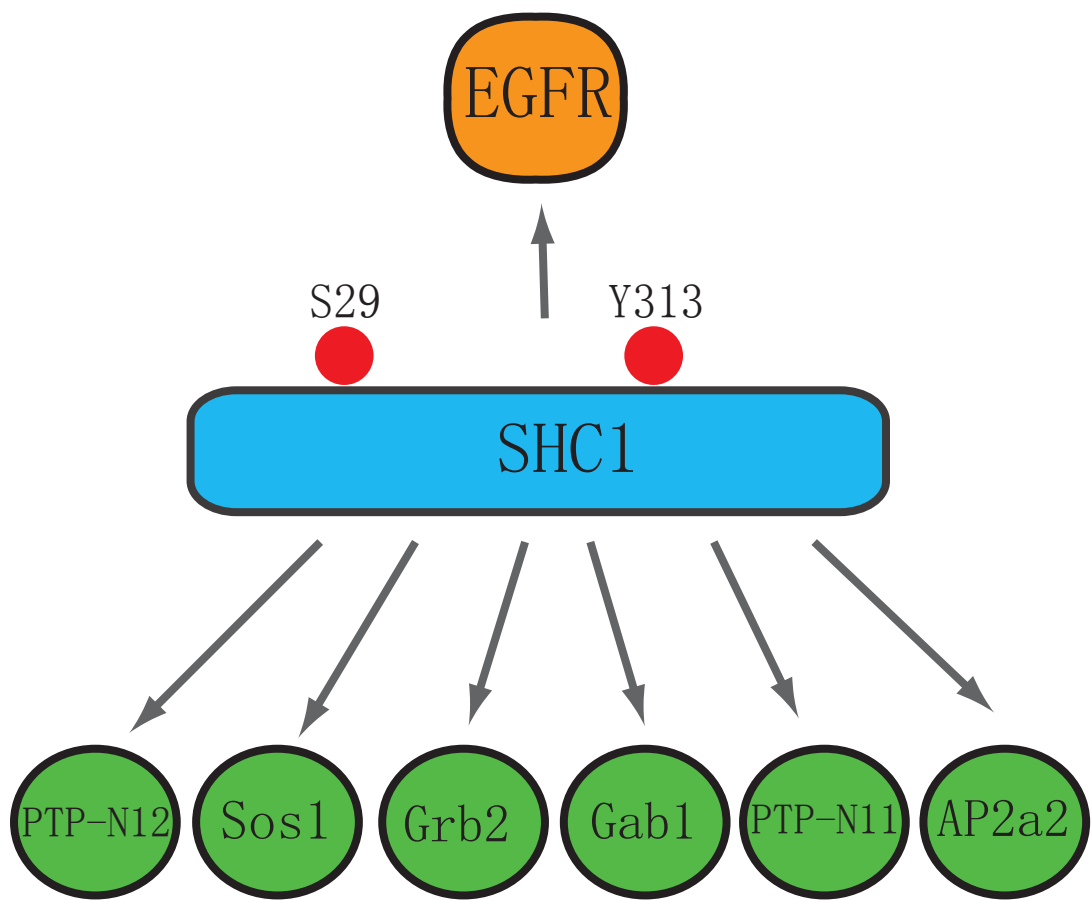

Supplement: Supplementary data 3 — Affinity maturation improves affinity capture, allowing detection of a core SHC1 signalling network. On-bead tryptic digestion and mass spectrometry analysis was carried out following immunoprecipitation from EGF-stimulated Rat2 fibroblasts, with the anti-SHC1 scFv 72_1A10. The identified members of the SHC1-mediated signalling complex are shown. See Supplementary Figure 2 for representative MS/MS spectra. Phosphorylated amino acids at positions 29 (S29) and 313 (Y313) of SHC1 were also detected and shown as red dots. [file mmc3.pdf]
