# Supplementary material for: Mapping protein interactions by combining antibody affinity maturation and mass spectrometry
Source: Anal Biochem. 2011 Oct 1;417(1):25–35. doi: 10.1016/j.ab.2011.05.005 (PMC3171153; doi:10.1016/j.ab.2011.05.005)
Supplement: Supplementary data 2 — Representative MS / MS spectra from selected peptides of SHC1 and its binding partners are shown. Corresponding peptide sequences, m/z values, and charge states are listed for each spectrum. [file mmc2.ppt]

## Slide 1
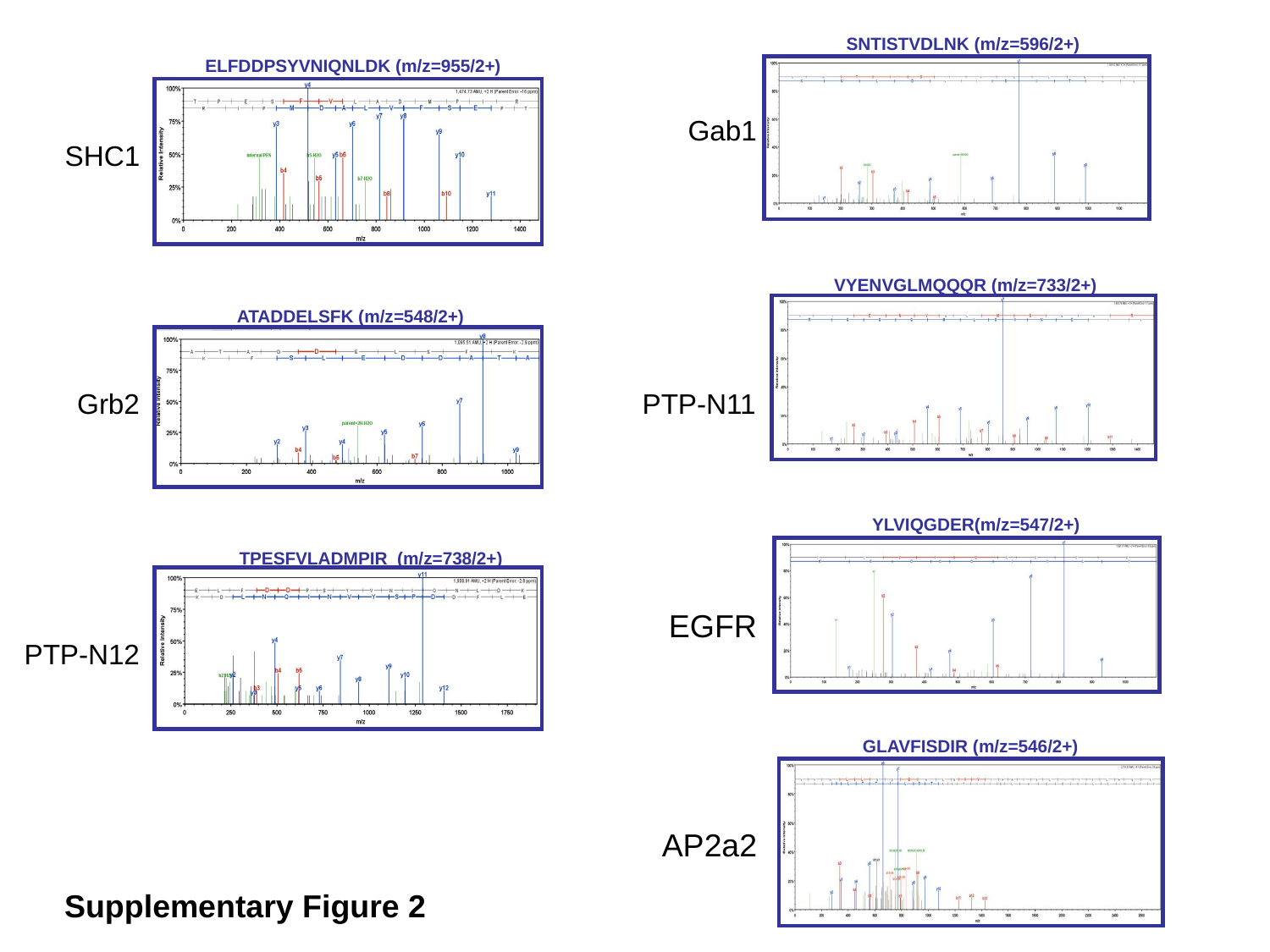

SNTISTVDLNK (m/z=596/2+)
ELFDDPSYVNIQNLDK (m/z=955/2+)
Gab1
SHC1
VYENVGLMQQQR (m/z=733/2+)
ATADDELSFK (m/z=548/2+)
Grb2
PTP-N11
YLVIQGDER(m/z=547/2+)
TPESFVLADMPIR (m/z=738/2+)
EGFR
PTP-N12
GLAVFISDIR (m/z=546/2+)
AP2a2
Supplementary Figure 2
