# Supplementary material for: Mapping protein interactions by combining antibody affinity maturation and mass spectrometry
Source: Anal Biochem. 2011 Oct 1;417(1):25–35. doi: 10.1016/j.ab.2011.05.005 (PMC3171153; doi:10.1016/j.ab.2011.05.005)
Supplement: Supplementary data 4 — A. SHC1 – Sequence analysis of primary clones (Selection 58) [file mmc4.doc]

**A. SHC1 – Sequence analysis of primary clones (Selection 58)**

| **Name** | **Clan** | **VH germline** | **VL germline** | **VH CDR3** | **VL CDR3** |
| --- | --- | --- | --- | --- | --- |
| 58_G05 x 2 | 3 | Vh3_DP-49_(3-30.5) | Vlambda3_DPL16_(3l) | ASAYGHYYYYYMDV | NSRDSSGNHLV |
| 58_E12 x 2 |  | Vh3_DP-49_(3-30.5) | Vk3_DPK21_(L2) | DIRGSYGGAFDI | QQYSNWPRT |
| 58_H12 |  | Vh1_DP-7_(1-46) | Vlambda6_6a | ETTFGGVIAY | QSYDSSNVV |
| 58_H02 x 4 |  | Vh3_DP-31_(3-09) | Vk1_DPK1_(O18,O8) | GGGYFDP | QQHKNFPRT |
| 58_G10 x 2 | 1 | Vh3_DP-49_(3-30.5) | Vlambda3_3h | GGLYGDLSWYFDL | QVWDRSSAHVV |
| 58_H05 x 4 |  | Vh3_DP-54_(3-07) | Vk1_DPK1_(O18,O8) | GKQLVRGFDP | QQHKNFPRT |
| 58_F12 |  | Vh1_DP-7_(1-46) | Vk1_L12 | GRALLNP | QQYNSYPLT |
| 58_F07 |  | Vh5_DP-73_(5-51) | Vlambda2_DPL12_(2e) | LRGVSAFDI | SSYAPRTNWV |
| 58_G09 |  | Vh1_DP-8,75_(1-02) | Vk1_DPK1_(O18,O8) | SATPNFDY | QQHKNFPRT |
| 58_G12 x 2 |  | Vh3_DP-49_(3-30.5) | Vlambda6_6a | SRPGGYADV | QSYDSRHWV |
| 58_E05 x 16 | 5 | Vh5_DP-73_(5-51) | Vlambda3_3j | STQWEQKGAFDI | QVWDSSSVI |
| 58_H09 |  | Vh1_DP-25_(1-03) | Vk3_L6 | TRYAPLLFDY | QQRSNWPYT |
| 58_G03 x 4 | 2 | Vh1_DP-14_(1-18) | Vk4_DPK24_(B3) | VHGGMDV | LQYYSSPFS |
| 58_E01 x 3 | 4 | Vh3_DP-49_(3-30.5) | Vlambda6_6a | VVVPAVHNYYYYGMDV | QSYDSSNWV |
| 58_E06 | 4 | Vh3_DP-49_(3-30.5) | Vlambda6_6a | VVVPAVHNYYYYGMDV | QSYDSSTWV |

**B. SHC1 – Sequence analysis of primary clones (Selection 72)**

**Clan 1 – VH germline Vh3_DP-49_(3-30.5), VH CDR3 sequence GGLYGDLSWYFDL**

| **Name** | **VL germline** | **VL CDR3** |
| --- | --- | --- |
| 72_1D11 | Vlambda3_3h | QVWNSTSDHVV |

**Clan 2 – VH germline Vh1_DP-14_(1-18), VH CDR3 sequence** VHGGMDV

| **Name** | **VL germline** | **VL CDR3** |
| --- | --- | --- |
| 72_1D06 | Vk1_DPK1_(O18,O8) | VNSMIISLLT |
| 72_1C06 | Vk2_DPK18_(A17) | MQGTYWPHT |

**Clan 3 – VH germline Vh3_DP-49_(3-30.5), VH CDR3 sequence ASAYGHYYYYYMDV**

| **Name** | **VL germline** | **VL CDR3** |
| --- | --- | --- |
| 72_1D09 | Vlambda1_DPL3_(1g) | AAWDDSLSAVV |
| 72_1C02 | Vlambda1_DPL2_(1c) | AAWDDSVKGVI |
| 72_3F05 | Vlambda3_DPL16_(3l) | NSRDSNGNLP |
| 72_1B11 | Vlambda3_DPL16_(3l) | NSRDSSGNHPV |
| 72_3H12 | Vlambda3_DPL16_(3l) | NSRDSSGNHVV |
| 72_3H02 | Vlambda3_DPL16_(3l) | NSRYSNGTLP |
| 72_1D07 | Vlambda3_3h | QLWDSSGDRVV |
| 72_2F04 | Vk1_DPK9_(O12,O2) | QRGSLR |
| 72_1D10 | Vlambda3_DPL16_(3l) | SSRDSSGNHVV |

**Clan 4 – VH germline Vh3_DP-49_(3-30.5), VH CDR3 sequence** VVVPAVHNYYYYGMDV

| **Name** | **VL germline** | **VL CDR3** |
| --- | --- | --- |
| 72_3H01 | Vlambda6_6a | HSYDARNHCM |
| 72_2G02 | Vlambda6_6a | QSFDTSNWV |
| 72_3F12 | Vlambda6_6a | QSYDGKNWV |
| 72_2E07 | Vlambda6_6a | QSYDNGDLSWV |
| 72_1B12 | Vlambda6_6a | QSYDSSWV |
| 72_3G09 | Vlambda6_6a | QSYDSSANWV |
| 72_2F08 | Vlambda6_6a | QSYDSSKWV |
| 72_1A10 x 14 | Vlambda6_6a | QSYDSSNWV |
| 72_2H07 | Vlambda6_6a | QSYDSSNGWV |
| 72_3H07 | Vlambda6_6a | QSYDSSNLNWV |
| 72_1B03 | Vlambda6_6a | QSYDSSPNWV |
| 72_2E01 x 8 | Vlambda6_6a | QSYDSSSWV |
| 72_3C09 x 2 | Vlambda6_6a | QSYDSSSHWV |
| 72_1B10 | Vlambda6_6a | QSYDSSTWV |
| 72_1D04 | Vlambda6_6a | QSYDSSTFWV |
| 72_3E08 | Vlambda6_6a | QSYDSSYHWV |
| 72_1B01 | Vlambda6_6a | QSYENGNWV |
| 72_1D03 x 2 | Vlambda6_6a | QSYGSSNHWV |
| 72_3C04 | Vlambda6_6a | QSYSSGTWV |

**Clan 5 – VH germline Vh5_DP-73_(5-51), VH CDR3 sequence STQWEQKGAFDI**

| **Name** | **VL germline** | **VL CDR3** |
| --- | --- | --- |
| 72_2G03 x 2 | Vlambda1_DPL3_(1g) | AAWDDSLNGWV |
| 72_2B01 | Vlambda1_DPL3_(1g) | AAWDDSLSASL |
| 72_1D08 | Vlambda1_DPL2_(1c) | AAWDDTLNYML |
| 72_1A01, 72_2C10, 72_3C11 x 49 , 72_2B09 x 5 | Vlambda3_3j | LVYDSDTGV |
| 72_3E09 | Vlambda3_3j | LVYDSGTGV |
| 72_3A09 x 2 | Vlambda3_3j | QAWDNSAAV |
| 72_3C07 | Vlambda3_3j | QAWDSGTGV |
| 72_3F01 | Vlambda3_3j | QAWDSNTGI |
| 72_2F07 | Vlambda3_3j | QAWDSSAVV |
| 72_3A06 x 3 | Vlambda3_3j | QAWDSSTAI |
| 72_2H11, 72_1B09 x 3 | Vlambda3_3j | QAWDSSTAV |
| 72_1C08 x 2 | Vlambda3_3j | QAWDSSTVV |
| 72_3H03 | Vlambda3_3j | QMWDSGTAV |
| 72_3E06 | Vlambda3_3j | QVCDSSTGV |
| 72_1D12 | Vlambda3_3j | QVWDSGTAV |
| 72_1A12 | Vlambda3_3j | QVWDSNAAV |
| 72_1C01 x 3 | Vlambda3_3j | QVWDSSSGV |
| 72_2E08, 72_1B06 x 5 | Vlambda3_3j | QVWDSSTAV |
| 72_2G08 x 2 | Vlambda3_3j | QVWDSSTGV |
| 72_1D05 | Vlambda3_3j | QVWDSSTVV |
| 72_1D02 | Vlambda3_3j | QVWDSSTWV |
| 72_2H09 | Vlambda3_3j | QVWDYSAAF |
| 72_3E02 | Vlambda3_3j | QVYDSDTGV |
| 72_1C07 x 5 | Vlambda1_DPL3_(1g) | VAWDDSLNSPV |
| 72_3H05 | Vlambda3_3j | QVWDSSAAV |
| 72_2E03 | Vlambda3_3j | LVYDSDTGV |
| 72_2E06 | Vlambda3_3j | QAWDSSTVV |
| 72_1A08 | Vlambda3_3j | LVYDSDTGV |

**Supplementary Table 1** SHC1 binding clones are grouped according to V_H_ and V_L_ CDR3 sequences. The primary unshuffled clones are shown in **Table A** which represents 47 sequences (15 uniques on CDR3 analysis and 21 uniques on whole scFv anlysis). **Table B**, representing the shuffled clones, is sub-divided according to V_H_ chain sequence giving clans 1 – 5 which represents 155 sequences with 59 uniques on CDR3 analysis and 64 uniques on whole scFv analysis
