# Supplementary material for: Mapping protein interactions by combining antibody affinity maturation and mass spectrometry
Source: Anal Biochem. 2011 Oct 1;417(1):25–35. doi: 10.1016/j.ab.2011.05.005 (PMC3171153; doi:10.1016/j.ab.2011.05.005)
Supplement: Supplementary data 5 — Sequences of selected anti-SHC1 scFv [file mmc5.doc]

**Supplementary Table 2. Sequence of chain shuffled clones**

**Clan 3**

heavy chain

(germline Vh3_DP-49_(3-30.5)) CDR1 CDR2 CDR3

58_G05 VQLVESGGGLVKPGGSLRLSCAASGFTFS__SYGMH__WVRQAPGKGLEWVA__VISYDGSNKYYADSVKG__RFTISRDNSKNTLYLQMNSLRAEDTAVYYCAK__ASAYGHYYYYYMDV__WGKGTLVTVSS

light chain

germline Vl3 (DPL16) CDR1 CDR2 CDR3 Kd (nM) k off (s^-1^)

58_G05 SSELTQDPTVSVALGQTVSITC__QGDSLRSYFAS__WYQQKPGQAPVLVIY__GKNNRPS__GIPDRFSGSSSGNTASLTITGAQAEDEADYYC__NSRDSSGNHLV__FGGGTKVTVLG 177 0.17

germline VK1_DPK9_02/02

72_2F04 DIQMTQSPSSLSASVGDRVTITC__RASQSISS__YLNWYQQKPGKAPKLLIY__AASSLQS__GVPSRFSGSGSGTDFTLTISSLQPEDFATYYC__QRGST__FGQGTRLEIKR 11.5 0.0526

**Clan 4**

Heavy chain

(germline Vh3_DP-49_(3-30.5)) CDR1 CDR2 CDR3

72_1A10 QVQLVESGGGVVQPGRSLRLSCAASGFTFS__SYGMH__WVRQAPGKGLEWVA__VISYDGSNKYYADSVKG__RFTISRDNSKNTLYLQMNSLRAEDTAVYYCAK__VVVPAVHNYYYYGMDV__WGQGTMVTVSS

Light chain

(germline Vlambda6_6a) CDR1 CDR2 CDR3 Kd (nM) k off (s^-1^ )

72_1A10 NFMLTQPHSVSESPGKTVTISC__TRSSGSIASNYVQ__WYQQRPGSAPTTVIY__EDNQRPS__GVPDRFSGSIDSSSNSASLTISGLKTEDEADYYC__QSYDSSNWV__FGGGTKLTVLG 25 0.081

**Clan 5**

heavy chain

(germline Vh5_DP-73_(5-51)) CDR1 CDR2 CDR3

058_E05 QVQLVESRAEVKKPGESLKISCKGSGYRFS__SHWIG__WVRQMPGKGLEWMG__IIYPGDSDTRYSPSFEG__QVTISADKSISTAYLQWSSLKASDTAMYYCAS__STQWEQKGAF__DIWGQGTMVTVSS

light chain

(germline λ 3_3j)

CDR1 CDR2 CDR3 Kd(nM) k off (s^-1^)

58_E05 SYELTQPLSVSVALGQTARITC__GGNNIGSKNVH__WYQQKPGQAPVLVIY__RDSNRPS__GIPERFSGSNSGNTATLTISRAQAGDEADYYC__QVWDSSSVI__FGGGTKVTVLG 452 0.219

72_1A01 -------------P----S--- ----------- ------------I-- ------- -------------------T------------ L-Y--DTLV ----------- 94 0.111

72_2E06 --- ----------- ------------I-- ------- -------------------------------- -A----T-V ----------- 56 0.0985

72_2B09 S--- -------H--- -------RPLSWSSI G Y---- ------------------------------F- I-Y--DTGV ----------- 56 0.0687

72_2C10 ---------------------- ----------- --------------- ------- -------------------------------- --------- ----------- 90 0.1057

(germline vλ1 (DPL3_(1g))

72_2B01 QSVLTQPPSVSGTPGQRVTISC__SGSSSNIGSHRVN__WYQHLPGTAPKLLIY__SNYHRPS__GVPGRFSGSKSGTSASLAISGLRSEDEADYYC__AAWDDSLSASL__FGGGTKVTVLG 7.6 0.0676

72_2G03 -R-------__--------RRY-H__---Q---A-------__RCSAAL___R--D------------------Q---------__-------NGWV__------L---- 14.3 0.0651
