# Supplementary material for: Mapping protein interactions by combining antibody affinity maturation and mass spectrometry
Source: Anal Biochem. 2011 Oct 1;417(1):25–35. doi: 10.1016/j.ab.2011.05.005 (PMC3171153; doi:10.1016/j.ab.2011.05.005)
Supplement: Supplementary data 6 — List of identified peptides for SHC1 and its binding proteins in anti-SHC1 scFv immunoprecipitation experiments [file mmc6.doc]

Supplementary Table 3
