# Supplementary material for: Mapping protein interactions by combining antibody affinity maturation and mass spectrometry
Source: Anal Biochem. 2011 Oct 1;417(1):25–35. doi: 10.1016/j.ab.2011.05.005 (PMC3171153; doi:10.1016/j.ab.2011.05.005)
Supplement: Supplementary data 7 — A minimum information about a protein affinity reagent (MIAPAR)[1] compliant document for anti-SHC1 single chain antibody scFv 72_1A10 [file mmc7.doc]

**Supplementary_Table 4 Minimum information about a protein affinity reagent (MIAPAR)[1] compliant document for anti-SHC1 single chain antibody scFv 72_1A10**

- 1. **Header**

| **Contact email** | md458@cam.ac.uk |
| --- | --- |
| **Contact Person** | Michael R. Dyson |
| **Organization** | Department of Biochemistry, Hopkins Building, Tennis Court Road, University of Cambridge, Cambridge, CB2 1QW, UK |

- 1. **Molecular entities**

| **Target** | **Description** | Human SHC (Src homology 2 domain containing) transforming protein 1 (SHC1) *Genbank id*: BC014158 |
| --- | --- | --- |
|  | **Production** | The SH2 domain of SHC1 was cloned, expressed and purified as described by Pershad *et al.*, 2010 [2]) |
|  | **Molecular characterization** | See (Pershad *et al.*, 2010 [2]) |

**Binder**

| **Binder** | **Description** | Single chain antibody (scFv) to the SH2 domain of SHC1  *Clone name*: 72_1A10  See (Dyson et al, 2011 [3]) |
| --- | --- | --- |
|  | **Production** | See detailed steps below |

**Binder Production:**

| **Step name** | **Materials** | **Reagents** | **Methods** |
| --- | --- | --- | --- |
| Filamentous phage display selection  *CV term:* PAR:0048 | McCafferty antibody phage display library[4] | See Schofield et al., 2007 [4] | Primary antibody phage display selection is described by Pershad et al., 2010 [2]. Affinity maturation by chain shuffling and reselection is described by Dyson et al., 2011 (submitted) |
| Sub-cloning scFv (CV term: PAR: 1057) into expression vector pSANG10 [5]  *CV term:* PAR:1154 | Expression plasmid pSANG10 [5]  CV term: PAR: 1207 | See Schofield et al., 2007 [4] | The polyclonal scFv round 2 output population was sub-cloned into the expression vector pSANG10 [5]. Clones were expressed in *E. coli* in 96-well plates as described previously [4] for screening. |
| Fluorescent immunoassay  CV term: PAR:1041 | Europium labelled anti-FLAG antibody [4]. | See Schofield et al., 2007 [4] | Screening performed by detecting binding of scFv in culture supernatants to antigen immobilized on 96-well polystyrene plates. scFv binding detected with Europium labeled anti – FLAG antibody in a DELFIA (dissociation-enhanced lanthanide fluorescent immunoassay) assay (Perkin Elmer) [4]. |
| scFv expression and purification  CV term: PAR: 0004 | Ni-NTA superflow resin (Qiagen 1018611) |  | scFv was expressed in *E. coli* BL21(DE3)and affinity purified as described previously from periplasmic extracts [5]. |

3. Binder/Target interaction

| **Binding description** | Specificity:  Specificity testing was performed for the parent scFv 58_E01 prior to affinity maturation [2] with no cross reactivity observed to a set of 20 related human SH2 domains.  Binding site:  The scFv binds the SH2 domain of human SHC1. The epitope is likely not involved in direct interaction with members of the EGFR signaling complex as judged by its ability to pull down members of this complex.  Binding constants:  k_d_ = 0.081 s^-1^, k_a_ = 3.2 x 10^6^ M^-1^s^-1^, K_D_ = 25.4 nM determined by surface plasmon resonance [3].  Selectivity:  Selectivity was demonstrated in the applications of immunoprecipitation and confocal microscopy.  Applications:  Immunoprecipitation [3].  Confocal microscopy [6] |
| --- | --- |
| **Experiment** | See detailed steps below |

| **Step name** | | **Materials** | **Reagents** | **Methods** | **Results** |
| --- | --- | --- | --- | --- | --- |
| Specificity  *CV term:*  PAR:1128 | SHC1  *Genbank id*: BC014158 | 20 human SH2 domains were expressed and purified as described previously [2]. Europium labelled anti-FLAG antibody [4]. | Europium labelled anti-FLAG antibody [4]. | scFv binding to its target and 20 related SH2 domains were assessed in a microplate DELFIA assay as described previously [2]. | Binding to the target (SH2 domain of SHC1) was observed but not the 20 homologous SH2 domains. |
| Affinity constant  CV term:  PAR:0646 | | The SH2 domain of SHC1 was expressed and purified as described previously [2]. | Buffer was filtered PBS (Dulbecco A, Oxoid, BR0014G) supplemented with 0.05% Tween20. | BIAcore surface Plasmon resonance (SPR) assay performed as described [3]. | Kinetic constants were determined as k_d_ = 0.081 s^-1^, k_a_ = 3.2 x 10^6^ M^-1^s^-1^.  Equilibrium dissociation constant was K_D_ = 25.4 nM. |

**Application test**

| **Application name** | **Materials** | **Reagents** | **Methods** | **Results** |
| --- | --- | --- | --- | --- |
| Immunoprecipitation and mass spectrometry  *CV term:* PAR:0069 | Anti-FLAG-M2 agarose was from Sigma (A2220) | The cell lysis buffer was 50 mM HEPES pH 8, 100 mM KCl, 0.5% NP40, 10% glycerol, 0.5 mM EGTA,1 mM Na3VO4, 2.5 mM NaPPi, 1 mM PMSF, protease inhibitors (Sigma) | A cell lysate was prepared from EGF stimulated Rat2 fibroblast cells, immunoprecipitation, on bead tryptic digestion and mass spectrometry analysis performed as described [3]. | A core EGFR signaling complex was identified [3]. |
| Confocal microscopy  CV term:  PAR:0663 | Anti-FLAG M2 antibody was from Sigma and Alexa555 Goat αmouse IgG was from Invitrogen. | As described by Colwill et al [6]. | Confocal images were generated of fixed MDCK cells expressing CFP-ErB2 and YFP-SHC1 that were stimulated with EGF for 5 or 15 mins. Anti-SHC1 scFv staining was detected with anti-FLAG mAb. | SHC1 co-localised with EGFR 5 and 15 mins post EGF stimulation in MDCK cells [6]. |

[1]J. Bourbeillon, S. Orchard, I. Benhar, C. Borrebaeck, A. De Daruvar, S. Dübel, R. Frank, F. Gibson, D. Gloriam, N. Haslam, T. Hiltker, I. Humphrey-Smith, M. Hust, D. Juncker, M. Koegl, Z. Konthur, B. Korn, S. Krobitsch, S. Muyldermans, P.A. Nygren, S. Palcy, B. Polic, H. Rodriguez, A. Sawyer, M. Schlapshy, M. Snyder, O. Stoevesandt, M.J. Taussig, M. Templin, M. Uhlen, S. Van Der Maarel, C. Wingren, H. Hermjakob, and D. Sherman, Minimum information about a protein affinity reagent (MIAPAR). Nature Biotechnology 28 650-653.

[2]K. Pershad, J.D. Pavlovic, S. Graslund, P. Nilsson, K. Colwill, A. Karatt-Vellatt, D.J. Schofield, M.R. Dyson, T. Pawson, B.K. Kay, and J. McCafferty, Generating a panel of highly specific antibodies to 20 human SH2 domains by phage display. Protein Engineering, Design and Selection (2010) gzq003.

[3]M.R. Dyson, Y. Zheng, C. Zhang, K. Colwill, K. Pershad, B.K. Kay, T. Pawson, and J. McCafferty, Mapping Protein Interactions by Combining Antibody Affinity Maturation and Mass Spectrometry. Anal Biochem submitted (2011).

[4]D. Schofield, A. Pope, V. Clementel, J. Buckell, S. Chapple, K. Clarke, J. Conquer, A. Crofts, S. Crowther, M. Dyson, G. Flack, G. Griffin, Y. Hooks, W. Howat, A. Kolb-Kokocinski, S. Kunze, C. Martin, G. Maslen, J. Mitchell, M. O'Sullivan, R. Perera, W. Roake, S.P. Shadbolt, K. Vincent, A. Warford, W. Wilson, J. Xie, J. Young, and J. McCafferty, Application of phage display to high throughput antibody generation and characterization. Genome Biology 8 (2007) R254.

[5]C. Martin, G. Rojas, J. Mitchell, K. Vincent, J. Wu, J. McCafferty, and D. Schofield, A simple vector system to improve performance and utilisation of recombinant antibodies. BMC Biotechnology 6 (2006) 46.

[6]K. Colwill, H. Persson, N.E. Jarvik, A. Wyrzucki, J. Wojcik, A. Koide, A.A. Kossiakoff, S. Koide, S. Sidhu, M.R. Dyson, K. Pershad, J.D. Pavlovic, A. Karatt-Vellatt, D.J. Schofield, B.K. Kay, J. McCafferty, M. Mersmann, S. Helmsing, M. Hust, S. Dübel, and S. Gräslund, A roadmap to generate renewable protein binders to the human proteome. Nature Methods submitted (2011).

ü
