# Supplementary material for: Mapping protein interactions by combining antibody affinity maturation and mass spectrometry
Source: Anal Biochem. 2011 Oct 1;417(1):25–35. doi: 10.1016/j.ab.2011.05.005 (PMC3171153; doi:10.1016/j.ab.2011.05.005)
Supplement: Supplementary data 8 [file mmc8.doc]

# Supplementary Methods

## Construction of pSANG10-HC

5’ – phosphorylated oligonucleotides 2270 (GGCCGCATCTCACCATCACCATCACCATCACCATCACCATCTGGTTCCGCGTGGTTCCATCGACACCTGGGTTTAATAAA) and 2271 (AGCTTTTATTAAACCCAGGTGTCGATGGAACCACGCGGAACCAGATGGTGATGGTGATGGTGATGGTGATGGTGAGATGC), which encoded a His10 – CLAMP tag [1], were annealed together in 100 µl of buffer PEI (20 mM Tris-HCl (pH7.5), 10 mM MgCl2, 50 mM NaCl, 1mM DTT) at a final concentration of 0.25 μM per oligonucleotide. This was achieved on a PCR block by denaturation at 95^o^C for 5 min followed by a slow ramp to room temperature. The annealed insert was directly ligated into the pSANG10-3F BamHI / HindIII cut vector [2], used to transform *E. coli* DH5α, plasmid DNA prepared and sequence confirmed (see **Supplementary File** pSANG10-HC.gb).

## Sub-cloning of scFv into pSANG10-HC

Inserts encoding selected scFv were prepared performing PCR amplification reactions with oligonucleotide primers pSANG10-pelB (CGCTGCCCAGCCGGCCATGG) and pSANG10_NotI_R (GATGGTGATGATGATGTGCGGATGCG) and template pSANG10-3F plasmid DNA harbouring selected scFv. Inserts were digested with NcoI / NotI and ligated into pSANG10-HC NcoI / NotI cut vector prior to transformation into the expression strain *E. coli* BL21 (DE3) V2R pRARE2.

[1]J. Huang, S.S. Nagy, A. Koide, R.S. Rock, and S. Koide, A Peptide Tag System for Facile Purification and Single-Molecule Immobilization. Biochemistry 48 (2009) 11834-11836.

[2]C. Martin, G. Rojas, J. Mitchell, K. Vincent, J. Wu, J. McCafferty, and D. Schofield, A simple vector system to improve performance and utilisation of recombinant antibodies. BMC Biotechnology 6 (2006) 46.
